# Constructing Fatality Review: A Policy Analysis of the Emergence of Domestic Homicide Reviews in England and Wales

**DOI:** 10.1177/10778012211068064

**Published:** 2021-12-23

**Authors:** James Rowlands

**Affiliations:** 1 Department of Sociology, School of Law, Politics and Sociology, 1948 University of Sussex, Brighton, UK

**Keywords:** domestic abuse, domestic homicide review, fatality review, policy analysis

## Abstract

In England and Wales, Domestic Homicide Reviews (DHRs) examine domestic abuse-related deaths to identify lessons to be learned. However, their emergence as a policy initiative has been little considered. To address this gap, a thematic discourse analysis of policy documents to 2011 was undertaken, examining the justification for, and conceptualization of, DHRs before their implementation. It is argued that DHRs were constructed as a taken-for-granted good, through which multi-agency partners would generate learning while the (gendered) subject was silenced. Attending to aspirations, contradictions, and tensions in the emergence of DHRs has implications for their understanding and operationalization in the present.

Domestic Violence Fatality Review (DVFR) seeks to understand domestic abuse-related deaths,^1^ usually intimate partner and sometimes familial, of which women constitute the majority of victims. Despite the differences between DVFR systems internationally (Dawson, 2017), they share a broadly similar framework. By identifying precursors to these deaths, capturing case data, and detecting gaps in service responses (Websdale, 2020), DVFR aims to improve responses to domestic abuse and prevent future deaths (Bugeja et al., 2017).

In England and Wales, DVFRs are known as Domestic Homicide Reviews (DHRs). Introduced in statute in 2004 but only implemented in 2011, DHRs can be described as a way to “illuminate the past to make the future safer” (Mullane, 2017, p. 261). DHRs’ explicatory potential has been reflected in scholarly interest, which has principally considered their findings. In contrast, little attention has been paid to policy discourse, with no analysis of DHR policy to date [albeit policy development areas have been identified, see Montique (2019); Mullane (2017); Neville and Sanders-McDonagh (2014); Sharp-Jeffs and Kelly (2016)].

This article addresses this gap by examining the emergence of DHRs, as represented in United Kingdom (UK) Government policy documents, to the point of their implementation in 2011. Examining policy discourse is an opportunity to consider the aspirations, contradictions, and tensions regarding the justification for the implementation of DHRs, as well as the conceptualization of their purpose. Such an examination attends to the conceptual basis of policy representations (Bacchi, 2009). However, the analysis of documentary policy discourse cannot interrogate the experiences of policy actors at the time, including their understanding(s), motivation(s), or decision-making process(es). Furthermore, while such an examination is valuable, it is retrospective; thus, consideration also needs to be given to how findings relate to the operationalization of DHRs since 2011.

Consequently, this article begins with a discussion of international DVFR practice, drawing attention to how DVFR is an example of increased attention to domestic abuse. The discussion then turns to DHRs in England and Wales, summarizing current knowledge. Thereafter, the methodology, a thematic discourse analysis of UK Government policy documents between 2002 and 2011, is described and the findings are presented. It is argued that policy discourse in this time can be read as constructing DHRs—regardless of the intention(s) or experience(s) of policy actors, and despite the subsequent contribution DHRs have made—as a taken-for-granted good, through which multi-agency partners would generate learning, while the (gendered) subject was largely silenced. The final part of this article discusses these findings both in their own right and in relation to issues and challenges in practice, policy, and research since 2011. In so doing, attention is drawn to the place of the (gendered) victim and how DHRs might be understood and operationalized in the present. Before concluding, study limitations and areas for further research are identified.

## The Emergence of DVFR Systems Internationally

To date, DVFR has largely developed in high-income, English-speaking countries; first in the United States (US) in the 1990s, before initially expanding to Australia, Canada, New Zealand, and the United Kingdom (where they are undertaken in England and Wales^2^) (Bugeja et al., 2017). A key contribution to the field is a volume edited by Dawson (2017), which summarizes the DVFR systems in these countries (including the UK, which is discussed as a case study below). Typically, accounts of DVFR systems describe their legislative and policy basis, and their operationalization (e.g., review panel formation and the cases considered), but tend not to address policy discourses per se. Despite this, evidentially, DVFRs come into being in a given jurisdiction due to a policy decision. Indeed, as a way of “counting”—a term describing not only tabulation, but also ways to account for and understand the lives of those killed, and to prevent future deaths—DVFR has a state imprimatur, unlike other forms such as femicide observatories (Walklate et al., 2020).

Recognizing that DVFR is a state-sanctioned tool raises the question of why this process has been adopted. In accounting for this, Websdale (2012) has described DVFR as a new type of democratic space that reinforces the rule of law, is a form of civic engagement, and has an institutional context. He also notes that DVFRs are found in functioning democracies (Websdale, 2020), arguing this means “however meagrely, [the state] might be willing to accommodate self-criticism and reflexivity” (Websdale, personal communication, May 22, 2020). Websdale also highlights factors like increased attention to domestic abuse, concern about crime and victims, and feminist-driven social/policy change.

Websdale's account does not address why DVFR has largely developed in high-income, English-speaking countries and this remains an area for further investigation (a shared heritage of English common law in these countries may be relevant). Yet, the factors Websdale highlights are present in the countries named above, including the impact of high-profile domestic homicide(s) (Dawson, 2017), changed responses to domestic abuse (Buzawa & Buzawa, 2017), and the influence of feminist activism (Htun & Weldon, 2018).

## A Case Study of DHRs in England and Wales

In England and Wales, similar factors can be identified and, following Websdale, might be implicated in the emergence of DHRs. Here, domestic abuse has undergone a significant change, as part of wider shifts to gender regimes and the public/private divide (Walby, 2011). More specifically, Payton et al. (2017) locate the origins of DHRs within the broader response to domestic abuse and identify several antecedents. This includes the development of different review systems (into police conduct, as well as the serious injury/death of children or vulnerable adults), in the same way that fatality reviews into child deaths were an important precursor to DVFRs. This also underscores Websdale's point about institutional context, given DHRs are but one type of statutory review process in the United Kingdom.^3^ Echoing Websdale's attention to high-profile cases in the emergence of DVFR, Payton et al. also identify the influence of media pressure and campaigning after high-profile killings, citing the murders of Julia and Will Pemberton in 2003, and Banaz Mahmod in 2006. The murders of the Pembertons are noted as being particularly significant, as their family secured a DHR even though the process had not been implemented (Walker et al., 2008). The subsequent “Pemberton Review” has had a formative impact (Monckton-Smith, 2012, p. 33; Websdale, 2010, p. 5), not least in terms of family status and specialist advocacy support.

DHRs were introduced in section 9 of the Domestic Violence, Crime and Victims Act 2004 [DVCV] and can be undertaken into deaths caused by a former or current intimate partner, family or household member, and deaths by suicide.^4^ To date, there have been three iterations of the statutory guidance that governs the DHR process (Home Office, 2011, 2013, 2016b). In the latest version, the purposes of DHRs are learning, acting on, and applying lessons learned from domestic homicide; preventing domestic abuse by improving service responses by intervening earlier; better understanding domestic abuse; and highlighting good practice (Home Office, 2016b, p. 6). Following a domestic abuse-related death, a DHR is commissioned by the relevant local Community Safety Partnerships (CSP).^5^ For each case, a DHR is conducted by an independent chair and a multi-agency review panel, leading to a report that is normally published anonymously (for an account, see Rowlands, 2020a).

However, although legislated in 2004, DHRs were not implemented until 2011, giving them a curious trajectory. Thus, while some CSPs undertook DHRs under their own auspices before 2011—the highest profile being the above-mentioned Pemberton Review—it was seven years before they became routine. Since then, reflecting the scale of domestic homicide, around 800 have been completed (Monckton-Smith, 2021, p. 215).

To date, the literature has primarily considered DHR case profiles, notably case circumstances, as well as the learning and recommendations produced (Chantler et al., 2020; Home Office, 2016a; Sharp-Jeffs & Kelly, 2016). DHR data have also been used to explore the experience of specific cohorts, including the experience of children (Stanley et al., 2019) and older people (Benbow et al., 2019).

Challenges with the DHR system have also been identified, albeit as observations made in the course of research rather than, for example, from a process evaluation per se. Issues include how decisions are made to conduct DHRs (Benbow et al., 2019); differences in participant status (Robinson et al., 2019); and concerns with system functioning (Montique, 2019; Neville & Sanders-McDonagh, 2014). The weakness of the UK Government's collation of findings, and the lack of a national repository, have been noted (Neville & Sanders-McDonagh, 2014; Rowlands, 2020a; Sharp-Jeffs & Kelly, 2016). There are also concerns about access to, and the quality of, DHRs (Bridger et al., 2017; Stanley et al., 2019). Finally, despite evidence of practice and policy change, the impact of DHRs remains unclear (Payton et al., 2017).^6^ However, despite these important contributions, there has been little examination of how DHRs function, both as a system or when conducted into specific deaths (Rowlands, 2020a).

Clearly, DHRs are being put to use, influencing practice, policy, and academic knowledge. Yet, there is a need to better understand the DHR system, and one way to do this is to consider its emergence. Thus, this article asks: How were DHRs discursively justified and conceptualized, and in so doing, what are the implications for their delivery in the present?

## Method

### Study Design

This article is an analysis of policy discourse, with discourses being historically contingent systems of meaning that are implicated in power/knowledge and which produce a “regime of truth” (Foucault & Rabinow, 1984, p. 73). Such regimes legitimize taken-for-granted assumptions about how a given problem is defined because certain ways of thinking are made possible and produce the object(s) with which they are concerned. Policy documents can be analyzed for how they explicate the available diagnosis and prognoses for a particular social problem although, as noted in the introduction, such an analysis does not attend to the intentions and experience of policy actors. Here, policy discourse analysis can consider how the problem (of domestic homicide) and the solution (DHRs) were represented. While there is not a formulaic method for discourse analysis (Willig, 2008), a “feminist genealogy” pays particular attention to how bodies are “represented, reproduced, regulated and restrained” (Pillow, 2003, p. 151). Similarly, this article is concerned with the body of the victim whose death is the trigger for, and anchoring moment of, a DHR.

### Data Collection

The study was a documentary (Bowen, 2009). The sampling frame was UK Government policy documents concerned with domestic abuse between 2002 and 2011. This period was chosen because 2002 was the earliest reference located in a policy document to DHRs, with 2011 being the year they were implemented. A total of 12 policy documents of various types and from two administrations were identified as providing a macro policy framing of DHRs and coded (see Table 1). Two further policy documents were excluded—a consultation about DHR implementation (Home Office, 2006a) and the first iteration of the statutory guidance (Home Office, 2011)—because they had a micro policy framing (i.e., they had a particularity of purpose and were technical). However, these documents were consulted, along with later versions of the statutory guidance (Home Office, 2013, 2016b), to inform the discussion of the findings regarding the operationalization of DHRs since 2011.

**Table 1.** Documents and Types of Analysis.

Author and year	Title	Type	Administration	Analysis
HM Government (2002)	*Justice for All*	White Paper	Labour	Coded
HM Government (2003)	*Safety and Justice*	Consultation	Labour	Coded
Home Office (2005)	*Domestic Violence: A National Report*	Report	Labour	Coded
Home Office (2006a)	*Guidance for Domestic Homicide Reviews under the DVCV 2004*	Consultation	Labour	Consulted
Home Office (2006b)	*National Domestic Violence Delivery Plan Progress Report 2005/06*	Report	Labour	Codes
Home Office (2007)	*National Domestic Violence Delivery Plan Annual Progress Report 2006/07*	Report	Labour	Coded
Home Office (2008)	*National Domestic Violence Delivery Plan Annual Progress Response 2007/08*	Report	Labour	Coded
HM Government (2009a)	*National Domestic Violence Delivery Plan: Annual Progress Report 2008/09*	Report	Labour	Coded
HM Government (2009c)	*Together We Can End Violence Against Women and Girls: A Consultation Document*	Consultation	Labour	Coded
HM Government (2009b)	*Together We Can End Violence Against Women and Girls: A Strategy*	Strategy	Labour	Coded
HM Government (2010)	*Call to End Violence against Women and Girls*	Strategy	Cons-Lib Coalition	Coded
HM Government (2011a)	*Call to End Violence against Women and Girls: Action Plan*	Report	Cons-Lib Coalition	Coded
HM Government (2011b)	*Call to End Violence against Women and Girls: Action Plan Progress Review*	Report	Cons-Lib Coalition	Coded
Home Office (2011)	*Multi-Agency Statutory Guidance For The Conduct Of Domestic Homicide Reviews*	Statutory Guidance	Cons-Lib Coalition	Consulted
Home Office (2013)	*Multi-agency Statutory Guidance for the Conduct of Domestic Homicide Reviews: Revised*	Statutory Guidance	Cons-Lib Coalition	Consulted
Home Office (2016b)	*Multi-agency Statutory Guidance for the Conduct of Domestic Homicide Reviews*	Statutory Guidance	Conservative	Consulted

The policy documents analyzed were read for content, and text relating to DHRs was extracted. Universally, these excerpts were small sections of text that dealt directly with DHRs. Given their size, excerpts were extracted inclusively, meaning the surrounding text was extracted if DHRs were presented as part of a larger account (e.g., where DHRs were discussed alongside other policy initiatives or intended outcomes). Additionally, text relating to homicide was extracted regardless of whether this was found alongside text related to DHRs or elsewhere (e.g., where the homicide rate was cited as an example of the prevalence and impact of domestic abuse). Text in appendices was also extracted, albeit these summarized the main text or represented the same in the form of an action plan.

### Data Analysis

A reflexive thematic analysis was undertaken, including data familiarization, coding, generating, reviewing, and then refining/defining and naming themes, and report production (Braun & Clarke, 2021). Reflecting the interest in discourse, thematic discourse analysis was then completed. That is, the themes identified were analyzed to identify any broader discourses (Botelle & Willott, 2020). Codes were generated inductively then grouped into themes. The analysis was iterative, with excerpts read and re-read to refine and clarify codes and themes. The analysis was conducted using qualitative data analysis software (NVivo^TM^), which facilitated the management of the data. The researcher remained responsible for the analysis and interpretation. The implications of this, regarding the researcher's positionality and trustworthiness of the research, are discussed further below.

Ethical approval was not required for the analysis of publicly available documents.

## Findings

The analysis identified how, in the policy documents to 2011, DHRs were justified as a policy response to a significant social problem (domestic homicide), with their purpose being conceptualized as to produce (system) learning. The analysis generated three themes that framed the justification and purpose of DHRs. First, DHRs were rendered as a taken-for-granted good, premised on state action which would both establish them and bring together multiple (most explicitly, statutory) agencies to undertake the process. Second, the victims of domestic homicide were the absent subject, being presented as the “Other” and, for the most part, rendered silent and denuded of subjectivity and agency. Third, the purpose of DHRs was to learn from, not about, victims of homicide. The following section addresses these themes, with Table 2 showing the occurrence of themes and sub-themes in the coded documents.

**Table 2.** Occurrence of Themes and Sub-Themes.

Themes and sub-themes	Files	References
**Learning the lessons**	11	53
Identifying gaps in responses	1	4
*Earlier reporting*	0	0
*Effective intervention*	1	1
*Sharing information*	1	1
*System failures*	1	1
Identifying risk factors	4	7
Prevention	10	33
*Agencies taking action*	7	10
*Avoiding future deaths*	8	12
*Improving practice*	2	2
*System change*	2	3
**Representation of victims**	10	36
Learning about victims	4	5
*Pemberton review*	1	1
*Perpetrator experience*	1	2
*Testimonial networks*	2	2
Learning from victims	5	7
*Agency contact*	4	6
*Case circumstance*	1	1
Making the case	6	23
*Numbers killed*	6	9
*Precursors*	2	4
*Typologies (intimate partner homicide)*	5	7
**Role of the state**	12	75
Establishment	12	38
*Implementation*	11	20
*Methodology*	1	4
*Statute*	8	10
Multi-agency partnership	10	28
*Statutory*	5	14
*Unspecified agencies*	9	11
Performance measurement	5	9

### The Role of the State (Including Multi-Agency Partnership)

In 2002, DHRs were first articulated as a statutory project, with proposals to legislate for “domestic violence murder reviews” (HM Government, 2002, p. 128, 132). In its power to establish and implement DHRs, the role of the state is manifest. Yet, across the excerpts, this intervention was rendered as benign, with DHRs being framed as a taken-for-granted good and without potential challenges. However, the state's power is neither untrammeled nor singular. Thus, as noted above, although legislated in 2004, DHRs were not implemented until 2011. No explanation for the delay was found, although in the excerpts there were suggestions of possible causes. A need to secure “clearance across government departments” and agree “governance arrangements” was noted (Home Office, 2007, p. 4). Reference was also made to “new burdens” for local government (Home Office, 2008, p. 35) and “on-going cross-Government work” (HM Government, 2009a, p. 30). While the delay was unexplained, the case for, and intention to implement, DHRs was oft-repeated (HM Government, 2009a, p. 30, 2009b, p. 70; Home Office, 2005, p. 19, 2007, p. 4, 2008, p. 35).^7^

Despite the state's central role, in the excerpts DHRs were not represented as the act of a singular sovereign state. Instead, their multi-agency nature was repeatedly stressed, although little explicated. On the first occasions where the agencies involved were described, the police were named alongside other unspecified agencies (HM Government, 2002, p. 132), with other statutory partners, notably health and social services, later being identified (Home Office, 2005, p. 19). The potential role of local authorities was also noted (Home Office, 2007, p. 20). Following this, for several years DHRs were little discussed in the excerpts. Where they were noted, DHRs were situated alongside the Coordinated Community Response (CCR) model (HM Government, 2009a; Home Office, 2007, 2008). The CCR, associated with Duluth, Minnesota, is a coordinated, multi-agency, and community-based response to domestic abuse.^8^ Within these broad references to partnership, there were explicit references to non-statutory agencies, including a reference to “voluntary agencies” as being one place where domestic abuse victims might disclose their experiences (Home Office, 2007, p. 14). There was also a reference to “local” agencies, with this juxtaposed against statutory ones (Home Office, 2008, p. 16). Given the framing of DHRs alongside the CCR, this is indicative of the potential role for voluntary agencies (in particular, domestic abuse services). The remaining texts refer in broad terms, such as agencies or partners (HM Government, 2009a, p. 30, 2009b, p. 70, 2009c, p. 19, 2010, p. 24, 2011a, p. 30, 2011b, p. 31).

In other words, accounts of multi-agency working were recurring but generalized. Where specified, multi-agency working explicitly referenced statutory agencies while the voluntary sector's role was largely nebulous. Moreover, multi-agency working—like the broader framing of state power—was narrowly rendered. That is, multi-agency participation in DHRs was represented as desirable, possible, and without potential challenges.

Additionally, from 2006 to 2009, a series of national delivery plans reported on progress against measures relating to domestic abuse, including a reduction in domestic abuse-related homicide (after 2007, this was formalized as National Indicator (NI) 34).^9^ This goal was situated as the primary, indeed singular, purpose of DHRs, premised on the lessons learned from case analysis being used to drive down the homicide rate.

### The Representation of Victims

Across the excerpts, victims of homicide were presented as an object of concern and, in aggregate, used as a rationale for action. Thus, the scale of domestic homicide justified action because “between a quarter and a third of victims of homicide are killed by a partner or former partner” (HM Government, 2002, p. 131). Indeed, “the figures on domestic violence homicide show the scale of the problem” (HM Government, 2003, p. 8).

Despite being the object of concern, victim agency and subjectivity were largely absent. As an example, there were no case illustrations in the excerpts; the only exception was in 2009 when the above-mentioned murders of Julia and Will Pemberton were noted. Yet, the case circumstances were not described and only the cipher “the Pemberton Review” was used. Moreover, while the publication of the Pemberton Review is noted, for unstated reasons it was described as “an exception rather than a template” (HM Government, 2009a, p. 30). Consequently, the case was—in policy terms—simultaneously exceptionalized and bounded.

The overlooking of victim subjectivity in the excerpts was underpinned by the de-mooring of victims from the relational and social milieu within which domestic homicide occurs. The focus of learning was orientated to what could be learned from victims based on agency interactions with victims before their deaths, rather than what could be learned about victim experience more broadly. Thus, there were few references to the potential of learning from a victim perspective (and thus the unique subjectivities at play). For example, an early reference drew attention to the “circumstances” of homicides (HM Government, 2002, p. 132), but with a concern for the response of agencies to risk. A similar orientation was found in a reference to the “the background” to killings (Home Office, 2005, p. 19).

Meanwhile, beyond agency interest, domestic homicide was little contextualized in the excerpts, with few references to victim subjectivity and agency, or individual or community dimensions. Indeed, the only explicit discussion of subjectivity (operationalized as “behaviour”) relates to the perpetrators of homicide (HM Government, 2009c, p. 19).^10^ There was also limited consideration of the role of those, other than agencies, who might illuminate victim agency and subjectivity, specifically the involvement of what Rowlands and Cook (2021) have called “testimonial networks” (i.e., family, friends, neighbors, and community members, and colleagues). There was, for example, only one reference to a victim's family, with their input noted but not explained (HM Government, 2009b, p. 70). Thereafter, no further references were found. Similarly, there was only a single reference to neighbors (HM Government, 2003, p. 37).

The nature of domestic homicide was also largely unstated, both in terms of its gendered profile and typology. Within the excerpts, the language was largely gender-neutral. Indeed, only four excerpts address the profile of the victims of domestic homicide. Yet, these references were not explicit, but rather artifacts of citational practice [by stating the number of women killed a week (Home Office, 2005, p. 27), the proportion of women and men killed by a former or current partner (Home Office, 2006b, p. 54, 2008, p. 42) or the proportion of women only (HM Government, 2009c, p. 34)]. As a result, the gendered nature of domestic homicide, whereby the majority of victims are women, was rendered invisible or at least muted. Likewise, men, the majority of perpetrators of domestic homicide, were also absent.

Regarding scope, none of the excerpts explicitly defined the cases to be considered by DHRs, although the implication, again by way of citational practice, was that DHRs would be concerned with intimate partner homicide. For example, of the two policy documents (HM Government, 2002, 2003) that preceded the DVCV, the first referred to intimate partner relationships. Of the 10 policy documents in the sample from after when DHR's legislative basis was in place, albeit unimplemented, four implied the scope of DHRs to be intimate partner homicide (by referencing victims being a former or current partner: HM Government, 2009c, p. 34; Home Office, 2005, p. 27, 2006b, p. 54, 2008, p. 42). As a result, adult family homicides, which have a different but also gendered profile, were overlooked.

### Learning the Lessons (Particularly About Risk)

Learning, to identify gaps in service responses and risk factors, was represented as central to the rationale for DHRs. This learning would allow preventative actions to be taken, with the goal of preventing future deaths. When implemented, DHRs would “ensure that the circumstances surrounding each domestic violence murders [sic] are reviewed” (HM Government, 2002, p. 132) and make it possible to “learn as much as possible from domestic violence homicides” (HM Government, 2003, p. 38). However, this learning potential was represented as being about risk and for agencies respectively, rather than, for example, an account of victim experience as a form of memorialization.

Across the excerpts, risk identification and performance management took center stage. DHRs were represented as a (criminal justice) tool that would “enable risk factors to be identified” (HM Government, 2002, p. 132). Meanwhile, pilots—in particular, in London (see Richards (2006)—were cited as having demonstrated the potential of DHRs, reporting on the importance of “information sharing and … highlight[ing] risk factors such as the correlation between domestic violence and child abuse” (HM Government, 2003, p. 38).

The rationale for this learning was that DHRs would enable the identification of learning (knowledge) to take action (power). After legislating for DHRs in 2004, the then Labour administration described DHRs as an opportunity for agencies who may have known about or suspected domestic violence to “look at the background and their [agency] involvement in each case, and learn lessons for the future” (Home Office, 2005, p. 19).

Across the excerpts, DHRs were directly implicated in power relations because it was assumed that they would inform the action of the state and its agents. Thus, learning would be preventative and “equip the police and other agencies to take action” (HM Government, 2002, p. 132), and “understand where systems failed, why the involvement of agencies or professionals did not lead to effective intervention, and what can be done to put the system right and avoid future deaths” (HM Government, 2003, p. 38). Indeed, as part of the CCR, DHRs would create opportunities to “successfully intervene” and prevent escalation (HM Government, 2009a, p. 12). In 2010, the new Conservative-Liberal Democratic Coalition called DHRs “an effective learning and prevention tool for local areas” (HM Government, 2010, p. 24). A year later, in the year of their implementation, the same government said DHRs would “support all agencies to identify the lessons that can be learnt …, with a view to improving practice and preventing future homicides” (HM Government, 2011b, p. 31).

## Discussion

Thus far, this article has argued for the need to attend to the emergence of DHRs as a policy initiative and has offered a reading as to how they were represented in policy documents to 2011, both in the rationalization for their introduction and conceptualization of purpose. Framing the rationalization and conceptualization of DHRs, three discourses were identified: the role of the state (including multi-agency partnership), the representation of victims, and learning the lessons (particularly about risk). The following discussion explores these discourses, and also draws upon them to reflect on the current understanding of DHRs, as well as contemporary policy and practice.

I turn first to the state. Although the state's role is implicit in the policy documents, it is unproblematized, and the decision to implement DHRs is represented as both a common-sense initiative and as a taken-for-granted good. True, the decision to implement DHRs could be seen as a positive step. As noted in the introduction, DHRs are a tool to better attend to domestic homicide and can be seen as an example of the success in moving domestic abuse from the private to the public domain (Walby, 2011). Yet, the state's record towards domestic abuse is mixed, at best, and it can be conceptualized as “enabling and constraining, as a potential ally and as an oppressive force” (Charles, 2000, p. 5). Such a nuanced assessment of the state's power is foreclosed in the policy documents. Given their authorship, to observe such a foreclosure is not surprising. Nonetheless, it is significant because it means the risks and opportunities of DHRs are left unrecognized. For example, Sheehy (2017) has argued that DVFR can be compromised because it can become a vehicle for state power (albeit her critique, which highlights the absence of feminist analysis in DVFR, is based on a small, solely documentary sample). This has contemporary relevance. To reiterate an earlier point, DHRs have been largely subject to secondary analysis. They have also often been represented uncritically (e.g., Monckton-Smith, 2012). As a result, scholarship to date has little engaged with this conflicted potentiality and scant attention has been paid to the intertwined promise and threat of DHRs, including the effect on their conduct and impact.

The state, though, need not be conceptualized as a singular entity; it is not an “it” but rather a terrain of powers and techniques (Brown, 1995, p. 174). Thus, the seven-year delay in the implementation of DHRs is of note. While the policy documents do not explain this delay, time would have been required to consult with stakeholders [as was done during this period (Home Office, 2006a)], agree on a methodology, and prepare statutory guidance. Although time-consuming, these steps would not account for a seven-year delay. While bureaucratic inertia may be an explanation, the references to the need for clearance and cross-government work, governance arrangements, and new burdens suggest that disagreement about financial/opportunity costs within government could have played a part. Additionally, DHRs were not the only measure in the DVCV to be delayed (House of Commons Home Affairs Committee, 2008, pp. 127–128). Regardless of the explanation, it is of note that, despite the then Labour administration positioning DHRs as a vital tool in the response to domestic abuse, and repeated statements of an intention to implement them, it failed to do so. Whatever the cause, such inaction is illustrative of the power of the state, a power that can be measured by what is not done as much as by what is done. Thus, the commitment to the introduction of DHRs in this period could be perceived as symbolic because there was a lack of an accompanying political will to overcome the blockage(s) to their implementation. This concern speaks to the present, with a contemporary illustration being the absence of a national repository 10 years after the introduction of DHRs. Arguably, the lack of a national repository has impeded the collation, analysis, and use of DHRs in aggregate, undermining their potential as a preventative tool.

The state's power is also capillary, represented in the policy texts with reference to multi-agency partnership. The call to partnership has an intuitive appeal, including in the response to domestic abuse, and has become a feature of the practice and policy landscape (Westmarland, 2012). This is evident in the excerpts which represent DHRs as a multi-agency mechanism, albeit largely a statutory one. Yet, partnership—conceptually, operationally, and in its outcome—is complex. While conditions for effective partnership have been identified (Hague, 2000), so too have potential barriers, not least in information sharing and inter-agency communication, and organizational and professional differences in understandings of risk and case management (Cleaver et al., 2019).

This raises several questions, including who a partnership is between, its operation, and its purpose(s) and outcome(s). Critically, while partnerships can appear effective, what can go unrecognized is that state agencies are favored, and non-governmental organizations face dilemmas associated with their participation or otherwise risk marginalization (Harvie & Manzi, 2011). For domestic abuse organizations, this can include conflict or compromise with their ethos and independence. At its core, this is a problematic of power differentials, an issue identified in the evaluations of some of the earliest domestic abuse multi-agency initiatives in the United Kingdom (Kelly, 1999). Critically, victims/survivors can also be put at risk by partnerships. Thus, Day and Gill (2020) explore the benefits of partnership working (which can enable domestic abuse support workers to advocate for victims/survivors in their encounter with the criminal justice system), but also its hazards (particularly for women with insecure immigration status, for whom contact with the police may be an additional threat).

In the context of DHRs, we might ask how do the review panels that deliver them operate? As with the role of the state, we might not be surprised that this question is foreclosed in the policy documents. But again, this absence means that the risks and opportunities of DHRs are left unrecognized, leaving a plethora of questions unasked. How do these panels function as a dialogical space within which multiple agencies participate and to which testimonial networks also contribute? How do those involved, individually and collectively, make sense of deaths and generate knowledge, and what discourses do they draw on in doing so? Furthermore, how does power operate and what dilemmas arise, not least for specialist domestic abuse services and members of testimonial networks? The import of such questions is not apparent in the policy documents as a partnership is rendered self-evident.

This is an issue in the present. The latest iteration of the statutory guidance notes that disputes and challenges occur in DHRs but does not suggest how to manage these, bar prescribing a need for resolution (Home Office, 2016b, p. 11). Meanwhile, what national training was once made available to support review panel members has long since withered, and researchers have little explored the inner workings of DHRs, including what is an enabler or barrier to participation (Rowlands, 2020a, p. 29).

Considering the representation of victims, there is also value in attending to both what is said and also silences in discourse. In the policy documents, gender, as well as victim subjectivity and agency, were largely absent.

The failure to recognize the gendered nature of domestic abuse has been a feature of UK Government policy. Thus, the UK Government's definition of domestic abuse has been criticized as obscuring gender (Kelly & Westmarland, 2014), with a similar charge leveled against the Domestic Abuse Bill 2020 (now the Domestic Abuse Act 2021) (Aldridge, 2021). By leaving unstated the gendered nature of domestic homicide, the policy documents provide a further example of this trend.

In addition, victim subjectivity (how someone lived and experienced their life) and agency (their capacity to make choices and act) are little recognized in the policy documents, both individually and with respect to the potential input of the wider community. The purpose of DHR was framed as to be to learn from, not about, victims of homicide. This distinction speaks to the orientation of the inquiry to the victim of homicide whose death is the focal point of a DHR. The former is extractive; the latter seeks situated knowledge. Illustrative of this tension is an imagined dialogue between a “battered” woman and a researcher, composed as a dialogic tool to explore DVFR. The former criticizes researchers who “feed off of women's blood and the homicide files put together by those professionals who turn up to deal with the mess and process it” (Websdale, 2005, p. 1198). While the risk of such “textual appropriation” is perhaps inevitable (Opie, 1992), the issue is to what extent this tension is recognized and counter-balanced in practice and research.

Indeed, a central challenge of a DHR is that it must grapple with the fact that death renders the subject silent. In the subject's place, a body remains which becomes a focus for discourse and practice (Troyer, 2020). Seen in this way, a DHR is a process in which a victim is scrutinized and re-signified as an object of investigation. However, this re-signification is not achieved through the voice of the victim, who can no longer speak, but rather through the DHR itself and the multiple actors within it, including professionals and members of testimonial networks. In many ways, the victims of domestic homicide are the absent referent of the policy documents, thereby “haunting” them [a term used by Gordon (1997) to consider that which is barely visible or not there and is so the “ghost” of the text]. This is a powerful metaphor for what Foucault called “subjugated knowledges,” that is, “naïve knowledges,” which are “beneath the required level of cognition and scientificity” (Foucault, 1980, p. 82).

As such, the subject is rendered an object and so becomes a case, being one of many objects to be cataloged. The challenge then is how we, following Ahmed (2019, p. 7), might “complicate” the use of a victim's experience. In unpacking the idea of use, Ahmed draws on the metaphor of a path, including the paths taken (because they are regularly used) and those that are not (and so requiring effort to use). To some extent, the operationalization of DHRs since 2011 has sought to address these tensions, encouraging review panels to go down paths that explore victim subjectivity and agency. Thus, the most recent statutory guidance says DHRs, by talking to professionals and members of testimonial networks, should “articulate the life through the eyes of the victim (and their children)” to “understand the victim's reality” (Home Office, 2016b, p. 7). Yet, despite the emphasis on victim voice, this is not, as described previously, explicitly a purpose of DHRs. Moreover, although an exemplar of a victim-focused narrative, the Pemberton Review is not referenced in any version of the statutory guidance, although it was cited as an example for writing DHRs (Home Office, 2012). This perhaps mirrors its exceptionalization and bounding in the policy documents. Finally, engaging with victim subjectivity and agency relies on how information is reported, but ironically some DHRs contain limited victim information (Bracewell et al., 2021).

Finally, we turn to the discourse of learning the lessons which are found throughout the policy documents. Drawing again on the idea of the paths most used, a recurring rationale for DHRs is the identification of learning (knowledge) to take action (power). This is evident in the policy documents, which position DHRs as a learning tool enabling intervention and the prevention of future homicides. Central to this framing is that the subjects of homicide can be cataloged and compared, in particular, to identify risk factors. This has implications for the conduct of DHRs, which—in the path they follow—may become more overly concerned with the identification and response to risk, despite limitations to this focus.

This is evident in the early concern in DHRs with making a finding as to the predictability and preventability of death, with this requirement found in the first two versions of statutory guidance (Home Office, 2011, p. 26, 2013, p. 27). Yet, despite the extensive policy and research evidence that has been developed around risk assessment, domestic homicide remains difficult to predict (Messing et al., 2021). Such a focus potentially forecloses other possibilities, given that a focus on risk alone does not consider a victim's experiences and death in their entirety, including the possibilities for broader learning. For example, focusing on identified high-risk cases (and particularly, incidents) occludes the daily realities of many victims/survivors of domestic abuse (Kelly & Westmarland, 2016). Meanwhile, a focus on learning from, rather than about, victims of domestic homicide (usually women) in the context of risk may simultaneously minimize their agency despite their being more than the object of a DHR. In effect, a focus on risk as a function of the outcome (the homicide) may be teleological in practice. This may work, potentially, to de-risk agencies if any analysis follows a simple causative chain that disregards consideration of victim subjectivity and agency (for a discussion, see Rowlands, 2020b).

This concern mirrors the broader literature, which focuses on DHRs as a tool to identify risk factors and inform safety planning (Richards, 2006). Indeed, as noted earlier, the homicide rate became a performance indicator (NI34). Such concern also reflects the broader preoccupation of policymakers with the classification and management of risk, including in policy around domestic abuse and homicide (Walklate & Hopkins, 2019).

To conclude the discussion of these three discourses—the role of the state (and multi-agency partnership), victim representation, and learning the lessons (about risk)—and their resonance with contemporary policy and practice, the concept of the examination is a way to articulate the challenges and opportunities of DHRs. For Foucault (1977/1991, pp. 187–192), the examination is a technology that “constitute[s] the individual as effect and object of power.” First, power is rendered invisible while the individual subject is foregrounded and treated as an object. Second, the individual is documented (i.e., they are described as an object). Third, the individual becomes a case that is an object of knowledge/power.

Using this conceptual framework, it is possible to attend to who or what is constituted as active, passive, and with what effect. The findings presented here suggest that, in policy documents to 2011, several troubling discourses can be identified. They represent the discursive formulation of a benign yet active state, marshaling agencies to produce learning that takes as its object victims of domestic homicide while denuding victims of agency/subjectivity. Collectively, these illustrate the potential tension(s) when making sense of domestic homicide, including between the subjective experience of those involved and the rational-legal discourses that may frame agency accounts and wider public policy.

However, as noted earlier, scholarly work has focused on the findings of DHRs. Consequently, DHRs have been largely treated uncritically, including the work of review panels, the generation of knowledge, and the implications of their use. Victims of homicide have also been treated passively, with little engagement with their representation. Left unrecognized is that DHRs are a site for the production of knowledge/power and are implicated in power relations, given that “it is not possible for power to be exercised without knowledge, it is impossible for knowledge not to engender power” (Foucault, 1980, p. 52).

### Implications for Practice and Policy

In the preceding discussion, it was argued that policy documents to 2011 constitute a discursive terrain about DHRs. One might ask as to the relevance of this study to the present. After all, DHRs were implemented in 2011. Moreover, as a process, DHR, like DVFR more generally, is an example of the changing response to domestic abuse and the increased attention being paid to domestic abuse (most predominately, the killing of women). While this is true, illuminating the discursive terrain in which DHRs emerged, including their rationalization and conceptualization, is valuable because policy guides actions (Pillow, 2003, p. 151). Engaging with the discourses in the policy texts analyzed also illuminates many contemporary challenges in practice, policy, and research. Most significantly, this includes the limited critical engagement with DHRs, including their doing and use. Without such critical engagement, the potential of DHRs may be constrained. At worst, DHRs may be damaging or, having a narrowed focus, be unable to change systems (Mullane, 2017).

Considering the circumstances of the emergence of DHRs helps us understand their use (and capacities) in the present. Perhaps the fundamental questions are as follows: Why do we undertake DHRs and for what purpose? To what extent has this changed between DHRs’ introduction and the present time, and what does this tell us about their limits and potential? Finally, what is the understanding of DHRs in their doing and how is this operationalized? Thus, are DHRs a means to generate knowledge to increase understanding of victim and perpetrator characteristics and risk profiles, and so improve system responses? In which case, should their focus be instrumental, concerned with agency or professional learning? Or should DHRs have a broader ambition, such as challenging the forensic narrative of domestic homicide (Monckton-Smith, 2012) and/or a form of memory justice (Walklate et al., 2020)? An answer to these questions is in part informed by the perspective one believes should be accessible to, or sought by, processes like DHRs, including whether they might provide a “wide-angled lens” on domestic homicide (Websdale, 2010, p. 5). Returning to the notion of a count described by Walklate et al. (2020), conceived as such, DHRs could offer a “thick” count where a victim's experiences are contextualized and situated within “structure, culture, time, and space.” Alternatively, a more procedural lens would be content with a “thin” count, focused on risk and with little consideration of victim subjectivity and agency, to the detriment of gender and other inequalities (Walklate et al., 2020, pp. 98–101).

The strongest illustration of contemporary relevance is how the issues identified in this article can be found in current policy and/or an assessment of the UK Government's stewardship of the DHR system. At the time of writing, the UK Government's Domestic Abuse Bill was progressing through Parliament. The articulation of DHRs during consultation on the Bill illustrates the resonance of questions about the place of homicide victims. In the consultation, rather than take center stage, victims haunt the description of purpose. DHRs are described as being so “*agencies and community organisations* can learn from shortcomings and improve their future response to domestic abuse” (HM Government, 2019, p. 81, emphasis added). Arguably, the commitments made—creating a public searchable repository, strengthening the learning process and the implementation of recommendations, learning from a process review in Wales, and sustaining advocacy services for bereaved families—are welcome but overdue, and unambitious after 10 years of DHRs. Moreover, if this study was repeated on policy documents since 2011, one would likely find these commitments are largely not new, illustrating a second phase to the curious trajectory of DHRs. In considering justification, purpose, and now delivery, this challenges us to ask why did DHRs capture policy interest initially but then failed to achieve sustained attention?

### Limitations and Future Research

This is the first study to examine policy discourses around DHRs, and I am an insider (DHRs are the subject of my doctoral study; I have also commissioned and chair them). My different experiences have led me to be troubled by policy representations of DHRs and concerned about the gap between policy and delivery. Thus, I have become increasingly cognizant of the benefits *and* challenges of DHRs, as well as their contingency as a meaning-making process (Rowlands, 2020b). However, these multiple perspectives also present a risk, given I might make assumptions about DHRs and/or my experiences may preclude me from considering other interpretations of the data. Given this and my use of policy discourse analysis, it would be remiss if my policy suggestions and representations were not also subject to (self) scrutiny (Bacchi, 2009). Consequently, the thick description offered here seeks to demonstrate trustworthiness by making my analysis “visible and verifiable” (Bowen, 2009, p. 38), while also enabling readers to make their own determination.

A limitation of this study is that it is based on a number of small excerpts extracted from a corpus of macro policy documents to 2011. As with Sheehy's analysis, which critiques DVFRs as “mere words on paper” (2017, p. 382) and is itself limited by its reliance on the same, this offers a partial perspective. Policy documents can be analyzed as artifacts, but cannot shed light on policy actor experience (including their understanding, motivations, or decision-making process), and so these could usefully be explored. An examination of macro policy documents since 2011, and the micro policy documents governing practice (principally, the statutory guidance), would also be valuable. Research is also needed into the operationalization of DHRs since 2011, including how the discourses identified here, and others, shape the practice and experience of those involved (including families) and the narration of individual deaths. Finally, as DHRs are part of a broader family of reviews, further research might consider connections with the other UK and international processes.

### Conclusion

This article presents a thematic discourse policy analysis of how DHRs have been framed in policy from their emergence to implementation, particularly their justification and stated purpose. Three discourses are reported concerning the role of the state (including multi-agency partnership), the representation of victims, and learning the lessons (particularly about risk). I have argued that the effect of these discourses is to foreground the state and its agents, while the victim and the perpetrator largely become de-gendered objects of concern who haunt the texts. Meanwhile, the policy solution—the DHR—is rendered as a taken-for-granted good. In reflecting on the findings reported here, I have also identified the implications for the capacities and limitations of DHRs in the present. Together, these pose challenges for practitioners, policymakers, and researchers. Most centrally, the question remains how to attend to the victim whose death is the trigger for, and anchoring moment of, a DHR in order to keep their subjectivity and agency in view.

## Notes

1. In both practice and in the literature, it is commonplace to refer to “domestic violence fatality reviews.” Consequently, I use this term when discussing these processes. However, to recognize the range of physical and non-physical harms which may precede death, I use “domestic abuse” more generally.

2. Northern Ireland has recently introduced DHRs (see https://www.nidirect.gov.uk/articles/domestic-homicide-reviews-dhrs). In Scotland “multi agency domestic homicide reviews” have been proposed (see https://www.gov.scot/policies/violence-against-women-and-girls/equally-safe-strategy/).

3. Other statutory reviews consider cases including homicides by patients being treated for mental illness, the death/serious harm of children or vulnerable adults, and deaths in custody.

4. DHRs into deaths by suicide are not further discussed because such cases are not referenced in the policy documents analyzed. Indeed, deaths by suicide are not explicitly referenced in section 9 of the DVCV (although the statute's language does not preclude them). It is of note that, over time, deaths by suicide have been brought into the scope of DHRs. Two years after the passage of the DVCV, a consultation into the implementation of DHRs noted that local areas “may also wish to carry out a review in cases of suicide” (Home Office, 2006a, p. 8). However, the first version of the statutory guidance did not specifically include deaths by suicide in the scope of DHRs. Instead, it referred to cases that “give rise to concern” which, even if they did not meet the criteria for a DHR, might be subject to some form of review (Home Office, 2011, p. 13). The next iteration of the statutory guidance clarified this, albeit still leaving deaths by suicide formally outside of the DHR process. It suggested that for deaths by suicide where the circumstances give rise to concern “the CSP should consider conducting a single agency individual management review or a smaller-scale audit” (Home Office, 2013, p. 14). The most recent iteration of the statutory guidance has brought deaths by suicide firmly into the DHR process and explicitly identified that such deaths, again where the circumstances give rise to concern, are in scope (Home Office, 2016b, p. 8). However, what constitutes a cause for concern is neither well-conceptualized nor defined. Consequently, there is likely considerable variation in interpretation and operationalization (for a discussion, see Rowlands, 2020a, p. 32). At the time of writing, there are no published data on the number of DHRs conducted into suicides.

5. CSPs, more formally known as Crime and Disorder Reduction Partnerships, bring together a range of local agencies and have a statutory responsibility for reducing crime and disorder, substance misuse, and re-offending in a local area.

6. It is beyond the scope of this article to explore if/how DHRs promote change; for a discussion see Rowlands (2020a). See Dawson (2017) for an international perspective.

7. By giving CSPs the responsibility for commissioning DHRs, they also became responsible for funding them. The largest direct cost in a DHR is the commissioning of an independent chair. Different funding models exist, which can include the CSP asking other statutory partners for contributions. However, in the author's experience and anecdotally, the cost is commonly borne in full or in a large part by the local community safety (and often, domestic abuse/violence against women and girls) budget.

8. In the United Kingdom, the charity Standing Together has produced guidance concerning the CCR (see https://www.standingtogether.org.uk/ccr-network).

9. National Indicators were announced by the UK Government in 2007 and introduced in 2008. They were used to measure performance by local authorities at the time.

10. Generally, beyond accounts of service contact and their abusive behavior, perpetrator experience is little explored in DHRs. This reflects process issues, including the often-limited participation of perpetrators and their family/friends in DHRs. It remains unclear if and how DHRs might illuminate pathways to becoming an abuser.
